# Chronic Hepatitis C Virus Infection Modulates the Transcriptional Profiles of CD4^+^ T Cells

**DOI:** 10.1155/2021/6689834

**Published:** 2021-03-12

**Authors:** Michal Holub, Alžběta Stráníková, Ondřej Beran, Simona Arientová, Oldřich Bartoš, Kateřina Kondelková, Stanislav Plíšek, Pavel Chalupa

**Affiliations:** ^1^Department of Infectious Diseases, First Faculty of Medicine, Charles University and Military University Hospital Prague, U Vojenské Nemocnice 1200, Praha 6 169 02, Czech Republic; ^2^Institute of Animal Physiology and Genetics, Laboratory of Fish Genetics, Czech Academy of Sciences, Liběchov, Czech Republic; ^3^Institute of Clinical Immunology, Faculty of Medicine in Hradec Králové, Charles University, Sokolská 581, Hradec Králové 500 05, Czech Republic; ^4^Department of Infectious Diseases, Faculty of Medicine, Charles University and University Hospital, Sokolská 581, Hradec Králové 500 05, Czech Republic; ^5^Department of Infectious and Tropical Diseases, First Faculty of Medicine, Charles University and University Hospital Bulovka, Budínova 2, Praha 8 180 81, Czech Republic

## Abstract

**Background:**

Chronic hepatitis C (CHC) is associated with altered cell-mediated immune response.

**Objective:**

The aim of the study was to characterize functional alterations in CD4^+^ T cell subsets and myeloid-derived suppressor cells (MDSCs) during chronic hepatitis C virus (HCV) infection. *Methodology*. The expression levels of the lineage-defining transcriptional factors (TFs) T-bet, Gata3, Ror*γ*t, and Foxp3 in circulating CD4^+^ T cells and percentages of MDSCs in peripheral blood were evaluated in 33 patients with CHC, 31 persons, who had spontaneously cleared the HCV infection, and 30 healthy subjects. *Analysis*. The CD4+ T cells TFs T-bet (T-box expressed in T cells), Foxp3 (Forkhead box P3 transcription factor), Gata3 (Gata-binding protein 3), and Ror*γ*t (retinoic-acid-related orphan receptor gamma) and activation of CD8+ T cells, as well as percentages of MDSCs, were measured by multicolor flow cytometry after intracellular and surface staining of peripheral blood mononuclear cells with fluorescent monoclonal antibodies.

**Result:**

The patients with CHC had significantly lower percentages of CD4^+^ T cells expressing Ror*γ*t and Gata3 and higher percentages of Foxp3-expressing CD4^+^ T cells than healthy controls and persons who spontaneously cleared HCV infection. The ratios of T-bet^+^/Gata3^+^ and Foxp3^+^/Ror*γ*t^+^ CD4^+^ T cells were the highest in the patients with CHC. In the patients with CHC, the percentages of Gata3^+^ and Ror*γ*t^+^ CD4^+^ T cells and the percentages of T-bet^+^ CD4^+^ T cells and CD38^+^/HLA-DR^+^ CD8^+^ T cells demonstrated significant positive correlations. In addition, the percentage of CD38^+^/HLA-DR^+^ CD8^+^ T cells correlated negatively with the percentage of MDSCs.

**Conclusion:**

Chronic HCV infection is associated with downregulation of TFs Gata3 and Ror*γ*t polarizing CD4+ T cells into Th2 and Th17 phenotypes together with upregulation of Foxp3 responsible for induction of regulatory T cells suppressing immune response.

## 1. Introduction

Chronic hepatitis C (CHC) represents a serious health problem. The global prevalence of hepatitis C virus (HCV) infection was estimated to be 71.1 million viremic infections in 2015, with HCV genotypes 1 and 3 being the most prevalent (44% and 25%, respectively). Despite great progress achieved in recent years in the treatment of chronic HCV infection, CHC is still associated with many challenges. First, an important proportion of patients with CHC are from low- and middle-income countries with a dense population and limited access to expensive therapies with direct-acting antivirals (DAAs) that are highly efficient in the treatment of chronic HCV infection [1]. Second, HCV infection occurs asymptomatically in almost 85% of patients, and the diagnosis of CHC is often delayed, leading to the development of serious complications, including liver cirrhosis and hepatocellular carcinoma [2]. Finally, in developed countries, HCV infection is frequently associated with the use of intravenous drugs, and users are frequently unaware of HCV transmission routes, symptoms of infection, and availability of DAAs [3]. All of these factors support the continued necessity to develop an efficient vaccine, and an enhanced knowledge of immune responses associated with CHC may help in this effort. However, developing a B-lymphocyte-based vaccine is hindered by HCV diversity, and therefore, T-cell-based vaccines seem to be a good approach [4].

Regarding the T-cell-based vaccine development, understanding of T-cell-mediated immune responses in CHC is essential [5]. It has been well documented that a subset of CD4^+^ T cells, regulatory T cells (Tregs), which are responsible for immunologic unresponsiveness, is significantly elevated in the blood and liver of patients with CHC [6, 7]. Conversely, another subset of CD4^+^ T cells, interleukin- (IL-) 17-producing CD4^+^ T (Th17) cells, is associated with spontaneous clearance of acute HCV infection [8]. Similarly, the clearance of HCV infection can be promoted by a subset of CD4^+^ T cells, Th1 cells that produce interferon (IFN)-*γ* and IL-2, whereas induction of Th2 cells that produce IL-4, IL-5, IL-10, and IL-13 can lead to viral persistence and development of CHC [5]. It is well known that the lineage commitment of naive CD4^+^ T cells is regulated by encountered antigens and the cytokine milieu at the time of T-cell receptor engagement [9]. These signals and their combinations are critical for the expression of specific lineage-defining transcription factors (TFs) that are responsible for the polarization of naive CD4^+^ T cells into Th1 cells, Th2 cells, Th17 cells, or Tregs. For the shift to the particular CD4^+^ T-cell lineage, the expression of a specific master regulator TF is essential. The TF T-bet (T-box expressed in T cells) regulates the shift of naive CD4+ T cells into Th1 cells, Gata3 (Gata-binding protein 3) into Th2 cells, Ror*γ*t (Retinoic acid-related orphan receptor gamma) into Th17 cells, and Foxp3 (Forkhead box P3 transcription factor) into Tregs [10]. Although Th1 and Th17 responses are advantageous in acute viral infections, it is possible that during chronic infections, such as CHC, these CD4^+^ T cell responses are skewed to Th2 cells and Tregs, CD4^+^ T cell phenotypes that are less efficient for viral clearance [11]. Moreover, CHC is associated with elevated levels of myeloid-derived suppressor cells (MDSCs) and persistent activation of CD8^+^ T cells [6, 12].

Thus, the aim of this study was to analyze the expression of the lineage-defining TFs in circulating CD4^+^ T cells and the percentages of MDSCs in peripheral blood from patients with chronic genotype 1 HCV infection, persons with spontaneous HCV clearance, and healthy subjects. Additionally, the correlations among these cells and other immunological and virological parameters were evaluated.

## 2. Materials and Methods

### 2.1. Study Groups

This study was conducted at two departments of infectious diseases of two tertiary-care hospitals between 2013 and 2015. The study was approved by the local ethics committees in Prague (IRB00002721, Etická komise Fakultní nemocnice Bulovka, IRB No. 1, Biomedical) and Hradec Králové (IRB00007431, Etická komise Fakultní nemocnice Hradec Králové, IRB No. 1), and it was performed in compliance with the Helsinki Declaration (1996 revision). All study subjects were enrolled only if they expressed their consent by signing written informed consent.

We enrolled 33 patients with chronic genotype 1 HCV infection who attended the hepatology outpatient clinic of either department. The patients were included in the study only if (1) the chronic HCV infection was confirmed by real-time polymerase chain reaction (RT-PCR), (2) the patients were indicated for the antiviral treatment of CHC, and (3) there was no history of previous therapy of chronic HCV infection. The demographic and clinical data are shown in Table 1. For comparison, 31 HCV-seropositive persons, who had spontaneously cleared HCV infection (16 males and 15 females; median age 41.5 years, range 27–69 years), were enrolled. These persons were referred to the hepatology clinic of both departments to rule out CHC and were included in the study only if (1) HCV RNA in the blood was not detectable and (2) untreated acute HCV infection was documented in their medical records. Normal values of studied parameters were obtained from the control group of 30 HCV-seronegative healthy volunteers (17 males and 13 females; median age 32.5 years, range 22–62 years).

### 2.2. Laboratory Methods

Peripheral blood was collected in 7.5 mL S-Monovette tubes with Li-Heparin and 2.0 mL S-Monovette tubes with K3EDTA (both Sarstedt AG and Co, Nümbrecht, Germany) from each study participant.

Peripheral blood mononuclear cells (PBMCs) for TF expression analyses in CD4^+^ T cells and MDSC measurements were separated using density gradient centrifugation (Ficoll-Paque Plus, GE Healthcare Bio-Sciences AB, Sweden). The cells were washed two times with an RPMI medium 1640 (Invitrogen, Paisley, UK) and resuspended in the RPMI medium supplemented with 20% fetal bovine serum (FBS) (PAA Laboratories Gmbh, Cölbe, Germany). Resuspended cells were diluted and divided into aliquots, with 10% dimethyl sulfoxide (CryoSure-DMSO, WAK-Chemie Medical GmbH, Germany) used as cryopreservative. Cryotubes were placed in a freezing container with isopropanol in a freezer at −80°C. Samples were stored in liquid N_2_ for long-term storage. For the analyses, the cells were carefully thawed in a water bath at 37°C, washed two times with phosphate-buffered saline (D-PBS, Life Technologies Limited, Paisley, UK), and resuspended in PBS at a concentration of 1 × 10^6^ cells per mL. We used antibodies (CD3 PerCP-Cy5.5, CD4 PE-Cy7, CD8 APC-eFluor 780, Ror*γ*t APC, T-bet PE, and Gata3 AF488 (eBiosciences, Vienna, Austria)) for the TF staining and Foxp3 Staining Buffer Set (eBiosciences) for the intracellular staining protocol. We used CD33 PE, CD11b FITC, HLA-DR PE-Cy5, and CD14 PE-Cy7 (eBiosciences) antibodies for MDSC staining. MDSCs were defined as CD33^+^, CD11b^+^, CD14^+^, and HLA DR^low/neg^ cells.

The expression of activation markers on CD8^+^ T cells was measured immediately after blood sampling with the BD Multitest™ CD8/CD38/CD3/HLA-DR (BD Biosciences, San Jose, CA, USA) according to the original protocol using a BD FACSCanto™ II in Prague and a Navios™ (Beckman Coulter, Brea, CA, USA) in Hradec Králové. CD38^+^, HLA-DR^+^, and CD38^+^HLA-DR^+^ cells were gated as a percentage of CD8^+^ T lymphocytes. The data were evaluated using FlowJo software™ (Treestar Inc., Ashland, OR, USA).

The detection of HCV RNA in the serum was performed as follows. First, nucleic acid was extracted using the Prepito (PerkinElmer, Waltham, MA, USA) automated magnetic separation system and Chemagic Preito NA Body Fluid kit™ (Chemagen, Baesweiler, Germany). Subsequently, the HCV RNA was quantitated using an EliGene HCV LC CE™ kit (Elisabeth Pharmacon, Brno, Czech Republic). The RT-PCR 2.0 for gen-C™ and GEN-C 2.0 Hepatitis C Subtyping™ kits (both from Nuclear Laser Medicine S.r.l., Naples, Italy) were used to determine HCV genotypes and subtypes. In addition, the detection of anti-HCV antibodies was performed by 3rd-generation enzymatic immunoassay with chemiluminiscent detection on the Architect i2000 analyser (Abbott, Abbott Park, IL, USA).

### 2.3. Statistical Analysis

All statistical analyses were conducted by a certified biostatistician in R software (R Core Team 2019, Vienna, Austria). To compare individual groups of subjects among themselves in a pairwise manner, we applied two-way ANOVA followed by post hoc Tukey's honest significant difference test. Correlations among individual parameters were evaluated using Pearson's product moment correlation coefficient. In all cases, only a *p* value < 0.05 was assumed to be significant.

## 3. Results

**Figure 1:**
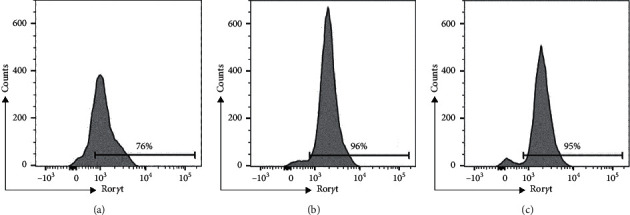
Representative histogram of Ror*γ*t expression in patients with chronic hepatitis C (a), persons with spontaneous viral clearance (b), and healthy controls (c).

Expression of the lineage-defining TFs in CD4^+^ T cells was significantly modulated by chronic HCV infection. Namely, the percentages of CD4^+^ T cells expressing Ror*γ*t and Gata3 were lower, and the percentage of Foxp3-expressing CD4^+^ T cells was higher in the patients with CHC in comparison to those in persons with spontaneous HCV clearance and healthy controls (Table 2). A representative histogram showing the differences in the Ror*γ*t expression level among the study cohorts is presented in Figure 1. Next, the patients with CHC had the highest ratios of T-bet^+^ to Gata3^+^ CD4^+^ T cells and Foxp3^+^ to Ror*γ*t^+^ CD4^+^ T cells (Table 2). The percentages of CD4^+^ T cells expressing Gata3 and Ror*γ*t positively correlated (*r* = 0.592, *p* < 0.001); similarly, the percentages of T-bet^+^ CD4^+^ T cells and CD38^+^/HL-DR^+^ CD8^+^ T cells demonstrated a positive correlation (*r* = 0.389, *p*=0.033). In addition, the percentage of circulating MDSCs correlated negatively with the percentage of CD38^+^/HLA-DR^+^ CD8^+^ T cells (*r* = −0.441, *p*=0.015).

## 4. Discussion

In this study, we evaluated the circulating levels of CD4^+^ T subsets (Th1, Th2, Th17, and Tregs), activated CD8^+^ T cells, and MDSCs in the peripheral blood of patients with CHC, persons with spontaneous HCV clearance, and healthy controls.

**Figure 2:**
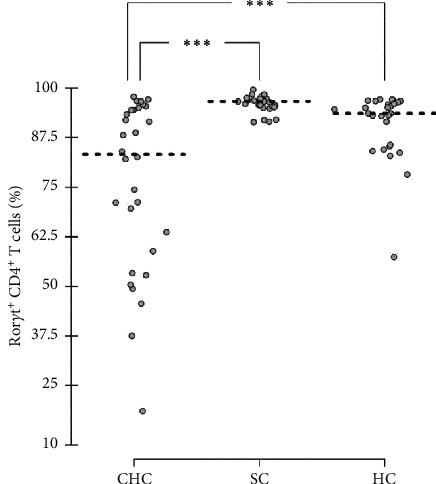
Percentage of CD4^+^ T cells expressing Ror*γ*t in patients with chronic hepatitis C (CHC), persons with spontaneous viral clearance (SC), and healthy controls (HC). ^*∗∗∗*^*p* < 0.001.

The decreased percentage of CD4^+^ Th17 cells observed in patients with CHC probably reflects a shift to more immunosuppressive response with Treg cells and MDSCs, a phenomenon that may be responsible for the persistence of HCV infection. It is well known that CD4^+^ Th17 cells have also been shown to play a role in viral infections. It has been speculated that the decrease in Th17 CD4^+^ T cells observed in the blood of HIV-positive patients is associated with the loss of mucosal barrier integrity [13]. Chronic HCV infection also modulates Th17 CD4^+^ T cell responses, which have beneficial and detrimental effects. Although the percentage of HCV-specific Th17 cells in the blood correlated positively with the severity of liver fibrosis and intrahepatic inflammation, a strong Th17 response may also lead to clearance of HCV infection [14]. Interestingly, the trend of the highest percentage of Ror*γ*t-expressing CD4^+^ T cells was observed in the study subjects with spontaneous HCV clearance, which, together with the finding of the decreased percentage of CD4^+^ T cells expressing Ror*γ*t in viremic patients (Figure 2), supports the suggested role of the Th17 response in HCV clearance. This phenomenon may have a genetic background because a study of two single-nucleotide polymorphisms located in the *RORC* gene demonstrated that the most common Th17-related *RORC* gene haplotype among the study population of Chinese Han females was associated with spontaneous HCV clearance [15]. On the other hand, intense Ror*γ*t expression in CD4^+^ T cells leading to the development of a robust Th17 response may aggravate inflammation, causing detrimental effects of CHC. It was suggested that this intense inflammation associated with autoimmune complications of CHC is elicited by HCV core antigen, which strongly augmented Ror*γ*t expression in naive CD4^+^ T cells in *in vitro* experiments [16]. This proinflammatory reaction is probably counterbalanced by Tregs, which can be reflected by the high ratio of Tregs to Th17 cells and increased frequencies of circulating Tregs observed in patients with CHC [6, 17].

The finding of the decreased percentage of Gata3-expressing CD4^+^ T cells in the blood of patients with CHC is surprising because persistent viral infections, including chronic HCV infection, are associated with a shift to a Th2 response and a simultaneous loss of Th1 CD4^+^ T cells [18]. Because Gata3 is able to induce the Th2 phenotype, its decreased expression may correlate with reduced Th2 responses. It is worth noting that the TF T-bet, which is responsible for Th1 polarization, directly modulates Gata3 expression [19]. Moreover, FoxP3 and Gata3 play a competitive role, similar to T-bet and Gata3 [20]. This notion can be supported by our findings of a high ratio of T-bet- and Gata3-expressing CD4^+^ T cells and by well-documented increases in the frequencies of circulating Tregs and Foxp3 expression in PBMCs in patients with CHC [6, 21]. Altogether, these data indicate enhanced polarization of CD4^+^ T cells toward Th1 and Treg phenotypes associated with a reduced Th2 response during persistent HCV infection. Despite the higher ratio of T-bet^+^/Gata3^+^ CD4^+^ T cells in the chronic HC patients, the frequency of T-bet-expressing CD4^+^ T cells did not differ among the study groups, indicating a reduced capacity for Th1 polarization. Since a strong Th1 response and activation of cytolytic CD8^+^ T cells are necessary for the clearance of HCV, reduced Th1 polarization can be an important mechanism leading to CHC.

Several correlations were observed among the study parameters. First, the positive correlation between the percentages of Ror*γ*t- and Gata3-expressing CD4^+^ T cells indicates their functional interplay. At low levels, Gata3 contributes to the development of Ror*γ*t-expressing CD4^+^ T cells; however, the role of Gata3 in Th1 or Th17 cells is still unclear [22]. Next, the observed positive correlation between T-bet-expressing CD4^+^ T cells and the activated CD8^+^ T cells, which express CD38 and HLA-DR molecules, reflects a direct connection between the Th1 response and cytotoxic immunity. It is well known that Th1 cells produce IFN-*γ*, inducing cytotoxic T cells that are crucial for the control of HCV replication and lysis of infected cells [23]. In addition to T-cell regulatory interactions, a negative correlation between monocytic MDSCs and activated CD8^+^ T cells found in the study subjects with CHC may reflect suppression of T-cell-mediated immunity by MDSCs. This notion is supported by the results of a study that demonstrated high levels of phosphorylated signal transducer and activator of transcription 3 and IL-10 in MDSCs isolated from patients with CHC [24]. When these MDSCs were depleted from isolated PBMCs, it led to *in vitro* restoration of T-cell-mediated immunity and significantly increased IFN-*γ* production. Thus, our findings likely suggest the immunosuppressive potential of HCV-induced MDSCs.

This study has several limitations. First, we did not evaluate the phenotypes of circulating CD4^+^ T cells using analysis of intracellular cytokines. Nevertheless, our findings are consistent with previous studies. Second, we did not provide detailed information on persons with spontaneous viral clearance, including when and how these subjects contracted HCV infection. The reason is that many of these subjects were drug addicts lost on follow-up, and retrieving additional clinical data from this cohort was impossible. Third, the study was originally designed as a longitudinal study of effects of IFN-based therapies of CHC on the frequencies of TF expression in CD4^+^ T cells for which the samples size was calculated. Therefore, the cohorts of CHC patients and persons with spontaneous viral clearance are relatively small. On the other hand, the study design enabled us to evaluate TFs profiles of CD4^+^ T cells that may play a role in the transition from acute to chronic viral infection.

## 5. Conclusions

The observed modulation of lineage-defining TFs in CD4^+^ T cells indicates downregulation of proinflammatory responses as an important mechanism leading to chronic HCV infection. Nevertheless, our data also suggest a protective role of the Th17 response, which can be utilized in the development of a preventive HCV vaccine.

## Figures and Tables

**Table 1:** Demographic and clinical characteristics of patients with chronic hepatitis C.

Parameters	
Age (years; range)^*∗*^	44 (25–68)
Male sex, total number (%)	20 (60.6)
HCV RNA (IU/mL)	2150000
Genotype 1b	27 (81.8)

^*∗*^Data are expressed as median; IU, international units.

**Table 2:** Comparison of the expression levels of transcriptional factors and the percentage of myeloid-derived suppressor cells among the study groups.

Parameters	CHC	SC	HC
T-bet^+^ CD4^+^ T cells (%)	15.95 (15.54)	12.3 (12.86)	14.9 (15.86)
Gata3^+^ CD4^+^ T cells (%)	56.05 (42.78)	68.6 (18.35)^*∗∗*^	65.4 (14.7)^*∗*^
Ror*γ*t^+^ CD4^+^ T cells (%)	83.30 (34.33)	96.6 (1.8)^*∗∗*^	93.6 (10.7)^*∗∗*^
Foxp3^+^ CD4^+^ T cells (%)	5.33 (1.9)	4.75 (1.08)^*∗*^	4.38 (1.16)^*∗∗*^
T-bet/Gata3	0.293 (0.444)	0.170 (0.181)^*∗*^	0.182 (0.254)^*∗*^
Foxp3/Ror*γ*t	0.077 (0.072)	0.049 (0.009)^*∗∗*^	0.048 (0.015)^*∗∗*^
MDSC (%)	0.86 (0.8)	0.66 (0.77)	0.91 (0.99)

Data are in medians (IQR); CHC, chronic hepatitis C; SC, spontaneous viral clearance; HC, healthy controls; ^*∗∗*^*p* < 0.01 vs. patients; ^*∗*^*p* < 0.05 vs. patients; MDSC, myeloid-derived suppressor cells.

## Data Availability

All data generated or analyzed during this study are included in this published article.
